# Cohort Profile: The Assessing Economic Transitions (ASSET) Study—A Community-Based Mixed-Methods Study of Economic Engagement among Inner-City Residents

**DOI:** 10.3390/ijerph191610456

**Published:** 2022-08-22

**Authors:** Lindsey Richardson, Anita Minh, Deb McCormack, Allison Laing, Skye Barbic, Kanna Hayashi, M.-J. Milloy, Kimberly R. Huyser, Kathleen Leahy, Johanna Li

**Affiliations:** 1 Department of Sociology, University of British Columbia, Vancouver, BC V6T 1Z1, Canada; 2 British Columbia Centre on Substance Use, 400-1045 Howe St., Vancouver, BC V6Z 2A9, Canada; 3 Department of Epidemiology, University of Washington, Seattle, WA 98195, USA; 4 Department of Occupational Science and Occupational Therapy, University of British Columbia, Vancouver, BC V6T 2B5, Canada; 5 Providence Research, 1190 Hornby, Vancouver, BC V6Z 2K5, Canada; 6 Faculty of Health Sciences, Simon Fraser University, Burnaby, BC V5A 1S6, Canada; 7 Department of Medicine, University of British Columbia, Vancouver, BC V5Z 1M9, Canada; 8 UBC Learning Exchange, University of British Columbia, 612 Main St., Vancouver, BC V6A 2V3, Canada; 9 EMBERS Eastside Works, 57 E Hastings St., Vancouver, BC V6A 0A7, Canada

**Keywords:** employment, income, people who use drugs, work, cohort study, mixed methods, qualitative, knowledge translation

## Abstract

The Assessing Economic Transitions (ASSET) study was established to identify relationships between economic engagement, health and well-being in inner-city populations given that research in this area is currently underdeveloped. This paper describes the objectives, design, and characteristics of the ASSET study cohort, an open prospective cohort which aims to provide data on opportunities for addressing economic engagement in an inner-city drug-using population in Vancouver, Canada. Participants complete interviewer-administered surveys quarterly. A subset of participants complete nested semi-structured qualitative interviews semi-annually. Between April 2019 and May 2022, the study enrolled 257 participants ages 19 years or older (median age: 51; 40% Indigenous, 11.6% non-Indigenous people of colour; 39% cis-gender women, 3.9% transgender, genderqueer, or two-spirit) and 41 qualitative participants. At baseline, all participants reported past daily drug use, with 27% currently using opioids daily, and 20% currently using stimulants daily. In the three months prior to baseline, more participants undertook informal income generation (75%) than formal employment (50%). Employed participants largely had casual jobs (42%) or jobs with part-time/varied hours (35%). Nested qualitative studies will focus on how inner-city populations experience economic engagement. The resulting evidence will inform policy and programmatic initiatives to address socioeconomic drivers of health and well-being.

## 1. Introduction

Amidst the interrelated public health emergencies of the drug toxicity crisis and the COVID-19 pandemic, the health equity impacts of the socioeconomic marginalization of vulnerable populations have emerged as a central concern [1,2,3,4]. A growing body of evidence identifies staggering individual, economic and community costs of unemployment, poverty, and material insecurity (e.g., food or housing insecurity) as key drivers of health harm among inner-city people who use drugs (PWUD), including non-fatal and fatal drug poisoning [5,6,7,8,9,10]. Labour market exclusion and employment instability are common experiences of socioeconomic marginalization in these populations, as indicated by high levels of volatility in accessing or having regular formal employment, particularly in the case of persistent, poly-substance or high-intensity substance use [11,12,13,14,15,16,17,18,19,20,21,22,23,24,25,26,27]. Due in part to a lack of accessible and viable formal employment opportunities, inner-city PWUDs are commonly relegated to informal, prohibited, or illegal income generation activities (e.g., informal recycling, sex work, drug dealing) that carry further risk of harm, including high-risk drug use, violence, criminal justice system involvement, disruption, or the discontinuation of substance use disorder treatment and overdose [8,9,15,28,29,30,31,32,33,34]. The consequences of labour market exclusion are of considerable concern in the context of the COVID-19 pandemic, given the exacerbation of the ongoing and unprecedented overdose epidemic amongst other health and social challenges facing PWUDs [35,36,37,38]. Upstream strategies to enhance the economic engagement and employment-related determinants of health inequity in drug-using populations are urgently needed. This paper describes the objectives, design, and characteristics of the Assessing Economic Transitions (ASSET) study cohort which aims to provide data on gaps and opportunities for addressing economic engagement in an inner-city drug-using population in Vancouver, Canada.

Existing population health research supports a bi-directional relationship between employment, work, and health [39], showing that both the availability of employment and quality of work influence wide-ranging health outcomes [40,41,42,43,44,45], and that health selects individuals out of employment or into specific kinds of employment [46,47,48,49]. However, most of this evidence has been drawn from representative or administrative data which may mask experiences of employment and economic engagement that are specific to disadvantaged populations [50,51]. More targeted studies, meanwhile, link suboptimal labour market engagement among inner-city PWUDs to higher disease morbidity and mortality [11,15,52,53,54], demonstrating that poverty and socioeconomic disadvantage are both causes and consequences of severe health harms in this population [8,52]. Still, the precise nature of employment and economic engagement in these populations, and their impact on the health and social well-being of inner-city populations, remain relatively less well understood [7,55].

**Figure 1:**
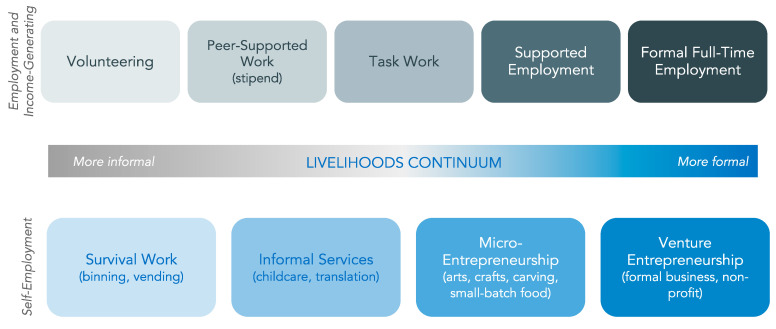
Community-generated livelihoods continuum.

These knowledge gaps are due to a dearth of data that adequately characterizes economic engagement and employment in inner-city residents. Critical to this research is specificity in measuring different dimensions of socioeconomic status, the intensity of work and economic engagement, the type of employment arrangement, and their relationship to diverse health outcomes [56]. As illustrated with a livelihoods continuum developed through community dialogues and consultations, the economic and income generation activities undertaken by community members and offered by community organizations range in both the type and intensity of economic involvement, span informal and formal labour markets and unpaid and paid work, and have implications for economic stability and health equity (see Figure 1) [57,58]. Economic activities may include survival-based activities involving the fulfillment of basic needs; unpaid volunteering through training and skills development opportunities; paid work or self-employment in informal, peer-supported and/or casual settings; as well as supported and full-market employment and entrepreneurship [57]. Importantly, community members move between different stages of the continuum in a non-linear way across the life course. In addition, individuals’ lifetime experiences of economic engagement and income generation along the livelihood continuum occur within a context of formal labour market restructuring that, since the 1970s, has resulted in the growth of working arrangements characterized by multiple dimensions of precarity—inadequate wages, unstable work-time agreements, and fewer protections for workers’ rights and benefits [40,42,59,60]. Studies applying concepts of precarious employment to the income generation activities of inner-city populations link their socioeconomic marginalization to the increased commodification of labour and of the weakening workplace, labour market, and social protections [61,62,63]. However, such studies have been predominantly limited to ‘peer work’ settings, work arrangements in which inner-city PWUDs are employed as consultants, knowledge brokers, and support workers in community-based research and clinical environments. As well, longitudinal measurements, with outcome measures designed and meaningful for PWUDs, are needed to disentangle the trajectories of work and health across the income generation spectrum over time [48,64]. Qualitative studies are also necessary to elucidate the experiences of work and health trajectories [52,65,66]. An evidence base that captures the range and quality of formal and informal economic engagement of inner-city residents over time is critical for understanding how labour market forces, social programs and protections, and socioeconomic marginalization interact to shape the health and well-being of these populations.

Additionally, there is a notable dearth of evidence about how, to what extent, and for whom intervention strategies may be effective at mitigating the experience of labour market exclusion and its associated harms. While socioeconomic disadvantage results from complex configurations of individual, social, structural and institutional factors [67], interventions are often implemented locally within a community context [68]. Sometimes referred to as low-threshold or supported employment opportunities, these innovative initiatives explicitly seek to facilitate economic engagement among marginalized populations by supporting individuals for whom full-time employment in the formal labour market may be inaccessible, inappropriate, or harmful. Models of low-threshold employment opportunities use both established and emerging models of economic engagement (e.g., accessible training programs, opportunity referrals, employment with flexible work arrangements, and community-based and supported work opportunities) to engage clients across the range of economic activities on the income-generation spectrum [69,70,71]. Cultural safety is relevant to understanding how these interventions may address the ongoing institutionalized racism that produces unequal socioeconomic and health burdens among Indigenous and non-Indigenous racialized peoples [72,73,74]. In the current global context, equity-based responses to the disproportionate impacts of the labour market disruption from the COVID-19 pandemic for disadvantaged populations will require targeted research on the extent to which such economic engagement models are able to mitigate these disparities [75].

We launched the ASSET study to fill these gaps in evidence and to aid in identifying programmatic and policy responses surrounding economic engagement and employment among inner-city, predominantly drug-using populations. This study uses a cohort-based, mixed methods examination of innovative, low-threshold economic engagement opportunities, coupled with an integrated knowledge translation and exchange (iKTE) strategy, as a platform for scientific, community, and decision-making purposes. It seeks to contribute an urgently needed evidence base assessing the relationship between innovative strategies that expand economic engagement access and health and well-being among socioeconomically marginalized individuals.

## 2. Cohort Description

### 2.1. Cohort Setting

The ASSET study cohort is based in Vancouver’s Downtown Eastside, an inner-city neighbourhood in Vancouver, Canada that is characterized by an open drug market, widespread poverty, as well as harm reduction advocacy and implementation, peer support organizations, and high community cohesion [76]. The community has also been identified as an epicenter of the ongoing drug poisoning (i.e., overdose) public health crisis [77]. It is amidst this unprecedented overdose morbidity and mortality that COVID-19 arrived [5,35]. The surge of non-fatal and fatal drug poisoning across Canada and the United States has been exacerbated by the pandemic through heightened isolation, and distress along with disturbances to unregulated drug markets, service provision, and to social support networks [37,38]. Research on clinical and harm-reduction interventions has shown effectiveness in reducing overdose-related mortality, but not the occurrence of overdose [78], emphasizing the urgent need for upstream preventive approaches [79]. Approaches that address the longstanding socioeconomic marginalization of inner-city residents and PWUDs have the potential to shift the social and environmental circumstances that underlie the risk of drug poisoning and associated harms [7].

A growing number of organizations have developed low-threshold employment opportunities to address the considerable harm associated with persistent socioeconomic marginalization common to many residents of the area. Examples include roles with task-based responsibilities providing flexibility, service provision validating community expertise, and the development of an economic hub that includes referrals, innovative opportunities, and retention supports. The development of viable models has produced an opportunity ecosystem to support economic engagement for highly disadvantaged individuals. However, little to no evidence on the impact of engagement with this opportunity ecosystem currently exists. Importantly, economic participation is dynamic and often involves more than one organization [80], rendering program-specific evaluations insufficient to assess the broader relationship between economic engagement and well-being. As such, this community-based study also operates as a research and evaluation umbrella across the economic engagement ecosystem, rather than an evaluation of a single organization or program in a context where there is widespread innovation in economic engagement opportunities for socioeconomically marginalized populations.

### 2.2. Objectives and Study Design

The key objectives of the study are to: (1) identify relationships between the different types and intensities of economic activity and individual health status and socioeconomic well-being; (2) examine the impacts of the COVID-19 pandemic and corresponding government responses on economic participation and associated dimensions of health and well-being; (3) explore how inner-city, drug-using populations navigate, perceive, and experience economic participation in relation to health and well-being; and (4) identify modifiable barriers to and facilitators of economic participation.

We use a parallel embedded mixed methods design to combine an open prospective cohort of inner-city Vancouver residents engaged in economic activity with nested longitudinal qualitative in-depth interviews among a subset of participants [81,82]. This design allows for: (1) quantitative assessment of the antecedents and consequences of economic activity, employment, and intervening forces; (2) in-depth analyses of the mechanisms and subjective experiences linking economic engagement and key outcomes; and (3) mixed methods analyses that merge analytic logics to identify potential points of intervention [81]. Qualitative and mixed-methods analyses will specifically allow for an in-depth understanding of the mechanisms connecting innovative economic models to outcomes, perceptions of economic models, participant experiences and unintended outcomes.

A central feature of the ASSET study is an integrated knowledge translation and exchange (iKTE) strategy that engages with multiple stakeholders, including affected community members who engage with low-threshold employment opportunities, local partner organizations who facilitate access to such opportunities, and government agencies with relevant programmatic and policy portfolios at local, regional, and national levels. This strategy aims to ensure that the evidence from this study is responsive and relevant to community needs by involving stakeholders at multiple points throughout the research process. All stakeholders serve as relationship and knowledge brokers to their respective organizations; have advised on the study’s conceptualization and on methodological best practices; and provide capacity and guidance for knowledge interpretation and dissemination. To date, stakeholder engagement through community consultations and steering committee meetings has been a key feature in the conceptualization of the income generation spectrum, the study’s research questions, study design, and the development of study instruments. Community members serve as peer research associates and, along with local community organizations, are engaged to support recruitment, outreach, the interpretation of findings, and the design of tailored iKTE dissemination materials. The study will merge participant data with organization-level information about different models of economic engagement to support organizations in refining their best practices, as well as their pandemic-specific adaptations. The ASSETS investigative team has regular update meetings with organizational and governmental representatives to inform ongoing study operations, data analysis and interpretation, and research and knowledge translation outputs. Through relevant stakeholder organizations and investigator-specific expertise, the study solicits culturally sensitive input and guidance on the stewardship, analysis, and interpretation of Indigenous and non-Indigenous racialized participant study data. The study findings will be disseminated through plain language summaries, study postcards, and knowledge syntheses to collaborators, community-based venues, and policymakers to support the uptake of results. Finally, the study will provide de-identified data back to the provider organizations for internal evaluation purposes. The provision of data will advance efforts to inform programmatic, policy, and budgetary decision-making, and support collaborating organizations’ funding, advocacy, and policy change efforts.

### 2.3. Eligibility and Recruitment

Eligible participants for the ASSET study: (1) are 19 years of age or older; (2) are residents of greater Vancouver; (3) are seeking or engaged in economic activity in the past three months as defined by the community-developed income generation spectrum [57]; (4) have had this activity verified through referral or study staff follow-up with an employer or provision of documentation of self-employment; (5) identify a past or present barrier to being in full-time employment; (6) provide written or verbal informed consent; (7) are willing to comply with study procedures; and (8) can communicate in English. Drug use is not an explicit inclusion criterion to allow for comparisons between drug- and non-drug-using participants, given employers’ and service providers’ goals to design low-threshold opportunities to support individuals regardless of their substance use patterns. Participants are ineligible for the study if they were deemed by trained study staff unable to provide informed consent due to intoxication, mental illness, or the inability to communicate.

The study uses a non-probability purposive sampling strategy that aims to enhance coverage of the survey sample across the income generation spectrum and socio-demographic dimensions of key relevance, including self-identified gender, ethnicity and housing dimensions. As in other studies that sample from populations that engage in illegal or illicit activity, we employed purposive sampling because conventional sampling and surveillance methods may be inappropriate for this population (i.e., no sampling frame exists) or may introduce bias [83]. Since April 2019, participants have been recruited through outreach strategies involving partner and affiliate organizations providing economic opportunities, information sessions, peer research associate outreach, and word of mouth. Outreach by study staff and peer research associates is a key part of this recruitment strategy and utilizes methods developed through longstanding research activities in this community. Peer research associates are community members employed by the study to support community engagement, the integration of localized knowledge, and to support community relationships. Targeted recruitment measures, such as focused engagement with women-serving organizations, are undertaken to ensure sufficient diversity in the sample. Participants are also recruited from ongoing research operations in the current study context: the Vancouver Injection Drug Users Study (VIDUS) and the AIDS Care Cohort to Evaluate Exposure to Survival Success (ACCESS), harmonized prospective cohorts of over 2000 community-recruited PWUDs in Vancouver [9,28,32,84]. Thus, while we intentionally did not set out to use sampling methods to derive a representative sample, our sampling strategies maximize the validity and relevance of evidence for the population of interest.

Eligibility for the ASSET study is verified in two steps. Eligibility is first verified with a 5–10 min interviewer-administered screening questionnaire either in-person at the study data collection site or by telephone. To protect the participants’ privacy, the screening questionnaire data are destroyed immediately after completion. The participants deemed ineligible are invited to contact the study team to be rescreened at a future date if their circumstances around eligibility change. The eligible participants are invited to book an appointment to complete the baseline study survey, at which point eligibility based on economic engagement is verified with either a referral from opportunity providers or documentation of self-employment, such as a business license or organizational materials, or other relevant documentation (e.g., paystub, email from employer or opportunity provider, confirmation of training enrollment). The eligible participants are provided with a consent form describing the study, planned follow-up assessments, and honoraria provided for participation, which is a $30 CAD stipend at the completion of each study visit, or $120 annually. The eligible participants are also asked to complete a locator form to gather their contact information for follow-up. Prior to the onset of the COVID-19 pandemic, informed consent was conducted in person before beginning the baseline interview. The consent processes were adapted during the pandemic to allow for remote consent provision that preserved physical distancing but retained stringent approaches used during in-person interviews.

We selected a subsample of survey participants for semi-annual longitudinal, nested qualitative interviews. This pre-planned explanatory sequential mixed-methods component was developed to center participant experiences and provide supplemental qualitative data to document the pathways and mechanisms connecting economic engagement to well-being emergent in the quantitative data [85]. Sampling for this component of the ASSET study used maximum variation quota-sampling, a technique that maximizes the range of perspectives captured in the quantitative data as well as representation across gender, ethnicity, and economic engagement type [86]. Recruitment for the qualitative interviews began in March 2021 and continued until saturation was achieved, with the initial qualitative recruitment ending in October 2021. Rolling enrolment in the qualitative arm of the study will continue, filling spaces if participants withdraw or are deceased. The participants provided separate written (during in-person activities) or verbal (during remote activities) informed consent for the qualitative assessment at the time of the qualitative interview. The qualitative participants receive a $30 honorarium for each qualitative interview.

### 2.4. Data Collection and Follow-Up

**Figure 2:**
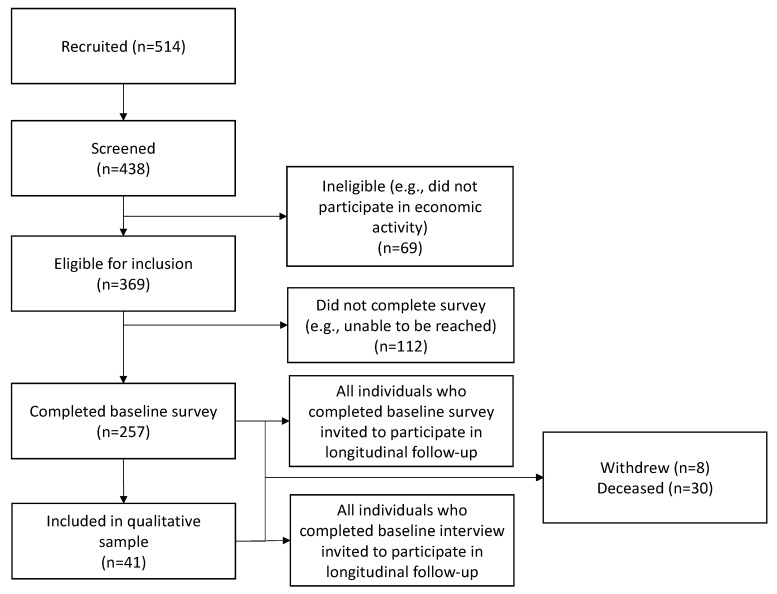
Screening and enrolment of the Assessing Economic Transitions (ASSET) Study cohort as of April 2022.

Consistent with the prospective cohort study design [87], the participants are followed up from baseline (i.e., the point of recruitment) until the end of the study, currently planned for October 2024, to optimize the assessment of changes over time (Figure 2). Follow-ups are completed quarterly to allow for the assessment of variation in economic engagement patterns, a schedule supported by the previous documentation of individuals cycling in and out of different economic activities [14,88], and to support participant recall [89]. Baseline surveys (60–90 min) and follow-ups (45–60 min) are completed using interviewer-administered questionnaires. These questionnaires adopted measures and an interview structure corresponding to a priori research questions developed through a review of the literature and longstanding research in the substantive area and research context, combined with key areas of substantive concern identified by economic engagement opportunity providers and community members. Where possible, the questionnaire drew on validated measures for a range of substantive areas of focus (e.g., employment precarity, food security, physical and mental health). The instrument was further validated through stakeholder consultation with community members and service providers, field-tested among prospective study participants, and refined prior to the initiation of formal recruitment.

The participants are followed using outreach and retention methods that promote interview completion and reduce loss to follow-up in the study population [90]. The outreach methods used by study staff include providing interview schedule cards and contacting participants prior to their due date to book an appointment via telephone, email, and mailed letters. For the harder-to-reach participants, the study staff also conduct physical outreach by dropping off letters at housing locations, local organizations, and health care providers. To maintain confidentiality, only outreach methods approved by the participants are used to contact them, and participation in the study is not disclosed. Extensive records are maintained and updated to identify the best methods to contact participants. Exit interviews are conducted with any participants who withdraw from the study to ascertain any final information they wish to convey, their reasons for withdrawing, and any feedback for the study they may have, though of note is that participants are more likely to be lost-to-follow-up than to formally withdraw. The survey data are gathered using the REDCap electronic data capture system and input directly into secure servers hosted at the University of British Columbia [91,92].

The qualitative interviews follow a semi-structured interview format, and topic guides were developed, as with quantitative instruments, based on previous research on employment among inner-city residents, a priori research questions, and adjustments based on interview piloting with community members. Bi-annual follow-up interviews capture the transitional experience in roles and types of economic engagement and employment, assets and disadvantages, the barriers and facilitators of economic engagement, the type and meaning of work, as well as overall health impacts. The qualitative participants lost-to-follow-up or withdrawing consent are prospectively replaced with demographically similar individuals.

While recruitment and data collection methods initially involved in-person procedures, we adapted the procedures for recruitment, follow-up data collection, and participant remuneration following the onset of the pandemic in March 2020. This resulted in a pause to data collection activities between March and July 2020, uniquely remote data collection between July 2020 and March 2022, and hybrid in-person and remote data collection from April 2022 onward. These changes were made to align with host institution guidelines, to accommodate participants’ needs, and to promote follow-up.

Recruitment efforts continued remotely through outreach to partner organizations, posters, letters, emails, phone calls, and other remote methods. Research staff conducted eligibility screening via telephone or by email, and the participants who conducted baseline interviews remotely were asked to email written informed consent for their participation. Prior to being interviewed, the participants were asked to confirm that they had access to a private space to be safely and confidentially interviewed. To support retention, outreach was conducted through letters, emails, phone calls, and other remote methods. Participant honoraria for the study were provided through cash payment by appointment, electronic transfer, direct deposit to their bank accounts, or alternative means as approved by trained study staff. 

All study activities are based at a store front field office centrally located in Vancouver’s Downtown Eastside and at research staff team members’ private residences during remote data collection. All study procedures have been approved by the University of British Columbia/Providence Health Care Research Ethics Board (H18-01858).

## 3. Study Measures

### 3.1. Survey Measures

The baseline, follow-up, and exit surveys collect data on sociodemographic factors (gender, ethnicity, housing status, relationship status, immigration status, education, and criminal justice system involvement), drug use and drug-related harms, social and economic exposures and outcomes, and health and well-being (see Appendix A). Baseline surveys collect additional data on lifetime experiences of drug-related harms, socioeconomic exposures and outcomes, and health. Given complex pathways between economic engagement and health that are often variable in relatively short periods of time, the ASSET study quarterly follow-up study visits collect participant data on economic engagement and employment precarity, material security, and drug-related harm prospectively. Follow-up surveys therefore focus on changes in participants’ experiences in the past three months.

Economic engagement is defined according to multiple dimensions, including: (a) category of IGA as delineated within the aforementioned community-developed livelihoods continuum of engagement in training, volunteer, peer-based, supported, or regular market employment [57]; informal, prohibited, or illegal income generation activities (e.g., bottle recycling, panhandling, sex work) [8]; (b) intensity of IGA, defined by the number of hours/week engaged in a specific activity; and (c) income, defined as IGA source-specific dollar amounts. Employment precarity is assessed using the validated PEPSO Employment Precarity Index [93]. Material security is measured using the modified Family Resource Scale [94], recently validated for use in the proposed study context [84]. Drug-related harm is measured according to high-risk drug use (e.g., injection, binge use, relapse), non-fatal overdose symptoms (ranging from mild to severe) [95], and the discontinuation of substance use disorder treatment.

Additional exposures of interest include: access to and characteristics of innovative service models for economic engagement (retention support, flexibility, security, individual agency in work decision making and referral access), all measured by community-developed instruments; exposures related to the pandemic including physical distancing directives (including self-isolation, avoidance of physical contact and indoor spaces); opportunity retention (question about loss of work due to COVID-19); receipt of COVID-19-specific government supports; and access to safe drug supply measures that were implemented during the early stages of the pandemic in the study context, including access to medically prescribed alternatives to street drugs [96]. Neighbourhood cohesion is measured through the validated Perceived Neighbourhood Social Cohesion Scale [97]. Additionally, data are collected on potential barriers to economic engagement such as criminal justice policy and criminal justice system involvement, employment-prohibitive addiction treatment regulations such as daily supervised ingestion of medications to treat opioid use disorder (MOUD), neighbourhood deprivation, life-altering events, as well as commonly gendered barriers (e.g., care responsibilities, engagement in sex work), and additional community-identified potential barriers to economic engagement (e.g., appearance-based discrimination) [14,27,52]. Data are also collected on access to the latent functions of employment (e.g., social contact and engagement in meaningful activity) given linkages with overall well-being as well as addiction treatment enrolment [98,99], which has in previous studies been shown to support entry into employment [14].

Additional outcomes of interest include: self-rated physical and mental health [100,101]; physical functionality (World Health Organization Disability Assessment Schedule) [102]; mental illness symptomology (Colorado symptom index [103], and Personal Well Being Index) [104]; exposure to violence [105]; and COVID-19 vaccine uptake.

### 3.2. Qualitative Concepts

The in-depth qualitative interviews aim to provide insight into concepts related to economic engagement-related assets and disadvantages, including beliefs, desires, and opportunities around employability; sociodemographic or socioeconomic circumstance; social capital, networks, and relationships; care responsibilities; and health status. The questions probe for concepts related to social-structural opportunities and constraints including stigma; criminalization; access to opportunities, services, and supports; and specific policies (e.g., earnings exceptions for income assistance recipients). The questions also elicit information key to the development of supportive engagement models, centring on emergent characteristics of low-barrier economic opportunities perceived by participants as facilitating engagement. These characteristics may include components of skills training, workplace and task-based flexibility, individualization, accessibility supports, opportunity referrals, or system navigation supports. Finally, the questions prompt for experiences of meaningful involvement in economic engagement as defined by the participant [106], and material improvements focusing on participant-defined enhancement in meeting physical needs.

### 3.3. Statistical Analyses

For the purposes of providing a cohort summary, we describe the standard baseline characteristics of the ASSET study sample. All analyses were conducted using R 4.2.0 [107].

## 4. Findings to Date

### 4.1. Recruitment and Retention

A flowchart summarizing participant recruitment, eligibility, and enrollment into the ASSET study between April 2019 and May 2022 is presented in Figure 2. Of the 514 people referred to the study, 438 were screened, 369 were eligible for inclusion, and 257 were enrolled. Reasons for ineligibility included having no connection to any organization offering economic or training opportunities in the past 3 months, not seeking or engaged in economic activity within the past 3 months, did not self-identify as having a barrier to employment, or being unable to provide informed consent. As of 2 May 2022, 257 participants had completed a total of 1654 study visits (257 baseline; 1347 follow-up), 8 participants had withdrawn, and 30 were deceased. Forty-one individuals have been recruited into the qualitative sub-study, completing 96 interviews to date (41 baseline; 55 follow-up). No qualitative participants have withdrawn and two were deceased.

Details for the quarterly and cumulative enrolment, follow-up, and retention are presented in Table 1. Across study periods, the average follow-up rate was 69.5%. As can be seen in Table 1, recruitment and retention rates were lower during the start of the COVID-19 pandemic. Non-informative missing data (e.g., refusal or invalid missing) were rare, with less than 6.2% of observations with invalid missing data across all key variables.

### 4.2. Cohort Characteristics

Table 2 shows baseline characteristics of the study sample. One participant’s data were removed due to concerns about their reliability. Of the remaining participants in the cohort, 57% identified as cis-gender men (*n* = 145), 39% as cis-gender women (*n* = 101), and 3.9% as transgender, genderqueer, or two-spirit (*n* = 10). The sample included participants who identified as Indigenous (Aboriginal, First Nations, Inuit, Metis; 40.0%, *n* = 99), Asian (Indian, Pakistani, Chinese, Vietnamese, Japanese, Filipino; 4.4%, *n* = 11), Black (African, Caribbean; 2.8%, *n* = 7), White (European or European descent; 49%, *n* = 122), and a race/ethnic background not captured by the above categories (e.g., Latin American, Middle Eastern; 4.4%, *n* = 11). Nine percent (*n* = 23) of the participants were born outside of Canada. The median age of the sample is 51 (IQR: 42–56). Most participants are single or dating (69%, *n* = 177). Most are stably housed (89%, *n* = 229), but 11% of the participants were homeless during the 3 months prior to their baseline survey (*n* = 27). Approximately half of the participants have less than a high school education (52%, *n* = 132).

Demographic characteristics across participants in the qualitative sub-study are similar to the overall cohort (see Appendix B). Of these, 46.3% identified as male (*n* = 19), 51.2% as cis-gender woman (*n* = 21) and 2.4% as transgender, genderqueer, or two-spirit (*n* = 1). The qualitative sample included Indigenous (34.1%, *n* = 14), Asian (9.8%, *n* = 4), Black (7.3%, *n* = 3), White (43.9%, *n* = 18), or a race/ethnicity not captured by the above categories (4.8%, *n* = 2).

### 4.3. Substance Use, Health, and Well-Being

As indicated in Table 3, substance use at baseline was highly prevalent in the sample, with almost all participants having used illicit drugs in their lives (99%, *n* = 254), and 86% of participants having used illicit drugs in the three months prior to their baseline interviews. Over three-quarters of the participants had engaged in binge use of illicit drugs during their lifetimes, defined as using more than usual for at least one day (78%, *n* = 195), and 32% had engaged in binge use in the three months prior to their baseline interviews. Almost all participants had engaged in daily substance use in their lifetimes (excluding alcohol and cannabis use), with 98% of participants who had used heroin or fentanyl daily (*n* = 251); and 96% of participants who had used stimulants such as cocaine, crack cocaine, or methamphetamine daily (*n* = 245). Daily heroin or fentanyl use in the three months prior to the baseline survey was reported by 27% of the sample (*n* = 69); and daily stimulant use by 20% of the sample (*n* = 51). Over half of the sample had experienced an accidental overdose during their lifetimes (56%, *n* = 140), and 12% had experienced an accidental overdose in the three months prior to their baseline surveys (*n* = 29). Half of the sample were enrolled in substance use disorder treatment (e.g., medications for opioid use disorder, alcohol and drug counselling, Alcoholics Anonymous, detox/recovery care, etc.) during the 3 months prior to their baseline surveys (*n* = 127).

The participants had a median score of 7.00 (IQR: 6.00, 9.00) on a scale assessing their satisfaction with their health (max score = 10.00), with higher scores indicating higher satisfaction. The participants had a median score of 14 (IQR: 6, 22) on the World Health Organization Disability Assessment Schedule (max score = 48) with higher scores indicating greater impairment. The participants had a median score of 12 (IQR: 5, 19) on a modified version of the Colorado Symptom index (max score = 50), with higher scores indicating higher mental health symptom frequency.

### 4.4. Economic Engagement

The economic engagement data at baseline are included in Table 4. Most participants had attended school or a training program beyond elementary or high school in their lifetimes (79%, *n* = 202), and 20% of the participants were currently enrolled in school or a training program or had plans to be enrolled at the time of their baseline surveys (*n* = 50). Over half of the participants had used employment services of some kind during their lifetimes (55%, *n* = 141), and 31% of the participants used employment services in the 3 months preceding their baseline surveys (*n* = 80).

The participants had several income sources. The participants received multiple forms of income assistance in their lifetimes, including employable or hardship income assistance (78%, *n* = 200), disability assistance (84%, *n* = 216), employment insurance (38%, *n* = 97), and old age security or public pension payments (8.6%, *n* = 22). Most participants received disability assistance in the three months prior to their baseline surveys (83%, *n* = 202). Almost all participants generated income through formal (96%, *n* = 246) and informal employment (90%, *n* = 245); over half the sample reported being self-employed during their lifetimes (*n* = 136, 53%). The most common informal sources of income generation were work arrangements in which participants received a stipend (75%, *n* = 192), and ‘binning’ which involves salvaging recyclable materials that may be exchanged for payment through municipal recycling programs (60%, *n* = 153). In the three months preceding their baseline surveys, more participants took part in informal income-generating activities (75%, *n* = 191) than in formal employment (50%, *n* = 129) or self-employment (20%, *n* = 50), with the most common of these activities being work arrangements that involved a stipend (59%, *n* = 152), and binning (31%, *n* = 80). As employees, the participants were largely employed as casual workers, doing on-call work or day-labour (42%, *n* = 105), or in permanent jobs with either part-time employment or varied hours from week to week (35%, *n* = 88). The participants had a median monthly income of $1955 (IQR: 1521, 2498) from all sources, including government assistance.

Over their lifetimes, 6.2% of the participants were either always or usually participating in the labor force, either always holding a job or looking for a job (*n* = 16), and 32% were usually holding a job or looking for a job (*n* = 82) (see Table 3). Thirty percent of the participants vary between working or looking for work and not working or looking for work (*n* = 78), and 26% were rarely participating in the labour force (*n* = 66). Approximately five percent of the participants have never taken part in the formal labour force (5.5%, *n* = 14), never having been employed or looked for formal employment.

Finally, the analysis of the qualitative data is not yet complete but will initially focus on experiences of innovative economic engagement models, including work flexibility, remuneration, supports and meaningful activity; dynamics surrounding key outcomes such as mental and physical health, economic well-being and the latent benefits of economic engagement; and individual, organizational, and structural barriers to economic engagement, with particular focus on policy constraints, engagement opportunities, stigma, and pandemic restrictions.

## 5. Discussion

The labour market exclusion of inner-city populations, including PWUDs, is intrinsically linked to the socioeconomic marginalization, material insecurity, and its population health impacts [11,15,52,53,54]. Emerging evidence suggests that the COVID-19 pandemic has been detrimental to the employment of these populations and has had exacerbating effects on the ongoing overdose epidemic [35,36,37,38]. There is emerging agreement that the disproportionate impacts of the intertwined drug toxicity and COVID-19 public health emergencies on marginalized populations must address their social and economic roots [1,3,4,79]. Yet, amidst a renewed focus on public health interventions, socio-economic well-being remains overlooked and understudied as an avenue for potential intervention to support the health and well-being of individuals facing barriers to economic engagement. We established the ASSET study cohort to provide urgently needed data on the relationship between economic engagement, drug-related harms, and health and well-being among an inner-city, predominantly drug-using population, conducted before and during the social and economic changes brought by the COVID-19 pandemic and public health and social welfare responses associated with the pandemic.

At present, the sample contains data from 256 socioeconomically marginalized participants followed for up to 3 years between April 2019 to May 2022, with follow-ups ongoing. As the baseline data presented here indicate, the sample reflects a population that experiences multiple forms of social and structural vulnerability. This ASSET cohort samples from a racially and ethnically diverse population of individuals engaging in economic activities in an inner-city neighbourhood in Vancouver, Canada. As in other studies of socioeconomically marginalized PWUDs [7,108,109], this sample has a higher representation of Indigenous and racialized individuals, resulting from the historical and systemic exclusion of racialized communities from adequate and high-quality economic opportunities [59,110]. Almost half of the participants have less than high school education. Consistent with other studies of PWUDs, there are also higher levels of mental health symptomology and disability in this population [103,111,112,113], reflecting broader gaps in the availability of workplace and public infrastructure to support labour market inclusion and accommodation for diverse health and ability needs. These characteristics, together with the composition of this sample by gender and migration background, suggest that this sample captures the experiences of population groups that have historically been excluded from conventional employment benefits and protections [63].

As the baseline data indicate, this population is also at high risk of drug-related harms. Drug use is not a criterion for inclusion in this study, which allows for this study to examine how economic engagement and employment are associated with current drug use when compared with no or infrequent drug use [11,15,52,53,54]. However, nearly all participants had engaged in high-intensity opioid or stimulant use in their lifetimes. Over half of the participants have experienced an accidental overdose in their lifetimes, and approximately one in eight participants in the 3 months prior to their baseline surveys. These estimates are comparable to the prevalence of overdose in Canada and among PWUDs globally [114], corroborating the risk presented by unpredictable concentrations of fentanyl and other synthetic opioids present in the current drug supply [77]. Only one in two participants currently access substance use disorder treatment. Whether our estimates represent an unmet treatment need, potential gaps in integrated service provision, the co-management of substance use and economic engagement, or some other relationship between treatment and economic engagement will be explored in subsequent analyses.

A key strength of the ASSET cohort study is the ascertainment of emerging models of employment and economic engagement as potential interventions to promote health and well-being. Most prior research has focused on samples engaged with peer-based work, or on narrow conceptualizations of work that eschew the range of ways that PWUDs generate income, and has limited potential for highlighting population-level barriers to economic engagement [61,62,63]. By contrast, the ASSETS cohort provides rich longitudinal quantitative and qualitative data across a range of exposures and outcomes including economic participation and employment, work intentions and barriers, material security, education and training, and service utilization and barriers. The baseline data suggest that a little more than half of the sample had accessed employment services in their lifetimes and three quarters currently engage in income generation activities through informal labour market participation. These estimates suggest a need to address gaps in labour market protections, expand opportunities for economic engagement, and recognize the value of alternative economic engagement in this population. Among employed participants, the baseline data indicate a high prevalence of casual, temporary employment, with varied or fewer working hours, and material inadequacy. These estimates indicate potential service and policy gaps in addressing material and social vulnerability faced by this population related to their labour market stratification and limited access to workers’ rights and social protections. The parallel embedded design of the study centres participants in corroborating and interpreting evidence about barriers and facilitators to the way that inner-city populations navigate, perceive, and experience economic participation [81,82]. The resulting quantitative and qualitative evidence has the potential to fill important gaps in existing knowledge about the conditions and experiences of work and the employment of marginalized inner-city populations.

Another strength of the ASSETS cohort data is the ability to support research on the structural and socioeconomic mechanisms that link the COVID-19 pandemic to population changes in health and substance use [37,38]. While it is widely understood that the pandemic and its associated economic and labour market shocks may widen existing social inequalities [115,116,117], its impact on the economic and income-generating activities of the disadvantaged population has not been well-documented. This longitudinal cohort has the capacity to fill this gap because it captures a range of experiences, involves data collected prior to and during the pandemic, and associated public health responses. For example, this cohort will capture information about how reduced access to opportunities and the necessarily curtailed activities of opportunity providers, a consequence of both the pandemic as well as longstanding inadequacy in social sector funding [118,119], affects the experiences of job loss and adaptations surrounding income generation, including the potential return to prohibited and illegal sources of income. These data may be used to examine whether and how these economic shifts at the individual level are associated with changes to individual psychosocial resources and support, substance use behaviours, physical health, and mental health and well-being [11,15,52,53,54]. These data may also capture how changing working conditions and expectations may be related to future economic engagement among marginalized populations. For example, these data may identify links between vaccine hesitancy and access to employment opportunities [120]. In so doing, these data have the capacity to go beyond documenting inequalities to understanding why they exist and potential avenues to address them.

Finally, the ASSETS study aims to examine organizational and institutional gaps and opportunities to support economic transitions for inner-city, drug-using populations. A necessary component of this research is a strong commitment to iKTE with policy makers, stakeholders and collaborators, service providers, and the affected community that requires ongoing coordinated attention. Collaborator community engagement has been and will continue to be a priority throughout the research process from conceptualization to dissemination to data sharing. This will maximize the data’s validity and their capacity to inform evidence-based policy and program decisions relevant to the participants and to application in other jurisdictions. Empirical evidence that links these innovations to health and social outcomes holds considerable potential to inform the development of sustainable, accessible alternative employment models of increasing relevance to populations that are socioeconomically and structurally marginalized.

This study has some important limitations. First, the COVID-19 pandemic began approximately a year after the initiation of the ASSET study, resulting in a temporary interruption of data collection activities as well as significant modifications to study procedures to move from in-person data collection to remote data collection. During the uncertain and rapidly evolving context of the pandemic, community partners and economic engagement opportunity providers were less accessible, opportunity structures shifted, and engagement with clients was severely curtailed. These circumstances limited our capacity for recruitment since we depended largely on our partner organizations’ engagement with their clients. The switch to remote data collection also limited our capacity to collect data since the requirement for participants to have access to remote technologies (i.e., phone, videoconferencing software) and private spaces for the interview may have limited our ability to engage with high-barriered participants. While this has clear consequences for recruitment and retention, we expanded our initial scientific aims and data collection instruments to incorporate the consideration of the pandemic’s consequences for our participants. Given that we started data collection prior to the pandemic, we are therefore uniquely positioned to assess pandemic-related impacts over time. Further, to minimise the impact to our study, we developed an infrastructure allowing for remote outreach and data collection operations including research tablets to support transitions between remote and in-person data collection.

An additional limitation is the use of self-reported measures that may be subject to social desirability or recall response biases [121,122,123]. This common concern with research among PWUDs has been met with multiple reports of a high reliability of responses [122,124,125,126], and our prior research has documented meaningful and significant relationships between self-reported income generation and health measures [8,9,15]. To minimize response biases, as in our previous work, we assure participants of the confidentiality of their responses, utilize experienced staff who share strong rapport with PWUDs and place sensitive questions toward the end of the study instruments.

Finally, this study may have limited generalizability. Nevertheless, the socioeconomic challenges of Downtown Eastside residents hold many similarities with other inner-city communities in Canada and internationally [127], pointing to the potentially widespread applicability of this research. A central scientific focus is to identify engagement characteristics that impact health and socioeconomic well-being, rather than specific jobs, providing important insights into the issues of employment measurement and developing evidence-based best practice guidance to support high transferability to praxis in other contexts [127].

### Future Plans

There are notable recent expansions of the ASSET study underway. As of April 2022, the ASSET study will begin another round of recruitment, aiming to add another 200 individuals to the cohort. This addition is expected to increase the sample’s representativeness and enhance statistical power for examining relationships between key exposure and outcome variables. An appropriate comparison group of individuals not engaged in ASSET-eligible economic activity will also be created via propensity score matching procedures to facilitate the examination of the effect of engagement with economic opportunity providers. These controls will come from the harmonized prospective VIDUS and ACCESS cohorts of community-recruited PWUDs in Vancouver. Select relevant outcomes for the ASSET study are also collected by these cohorts, alongside the identical measurement of most covariates, facilitating widespread comparisons.

## 6. Conclusions

By focusing on different types and intensities of economic activity and their relationships to individual health status and socioeconomic well-being in inner-city populations, including PWUDs, this cohort responds to several critical knowledge gaps. The data collected in this cohort enhance characterizations of employment and work-related dimensions of socioeconomic marginalization in multiply barriered populations. This cohort also supports research on the impact of the COVID-19 pandemic and corresponding government responses on economic participation and its associated relationship with health and well-being in inner-city populations. Nested qualitative studies will explore how inner-city, drug-using populations navigate, perceive, and experience economic participation in relation to health and well-being. Finally, this community-based study centres a comprehensive iKTE strategy within a research and evaluation umbrella across an economic engagement ecosystem. The evidence generated from this cohort will ultimately aid in identifying modifiable barriers to and facilitators of economic participation, and in doing so, inform urgently needed upstream interventions to support the health and well-being of inner-city populations. Importantly, efforts to address the health equity impacts of economic well-being require detailed understandings of population-specific dynamics and barriers surrounding economic engagement. The ASSET study offers an approach designed to harness innovation in community economic engagement to support improvements in equity across a range of understandings of health and well-being.

## Figures and Tables

**Table 1:** Quarterly and cumulative enrolment for the quantitative sample, follow-up, and retention from April 2019 to April 2022.

	Baseline Visits	Cumulative Enrolment	Eligible for Follow-Up	Follow-Up Visits	Withdrew	Deceased	Follow-Up Rate (%)
Round 00 (Apr–Jul 2019)	95	95	0	0	2	0	NA
Round 01 (Jul–Oct 2019)	87	182	93	84	1	2	90.32
Round 02 (Nov 2019–Jan 2020)	24	206	177	155	0	4	87.57
Round 03 (Feb–Apr 2020)	20 *	226	197	80 *	0	0	40.61 *
Round 04 (Apr–Jul 2020)	0 *	0 *	0 *	0 *	0 *	0 *	0 *
Round 05 (Jul–Oct 2020)	0	226	217	153	0	4	70.51
Round 06 (Nov 2020–Jan 2021)	11	237	214	144	2	2	67.29
Round 07 (Feb–Apr 2021)	4	241	221	149	1	3	67.42
Round 08 (Apr–Jul 2021)	4	245	221	147	2	5	66.52
Round 09 (Jul–Oct 2021)	5	250	218	136	0	3	62.39
Round 10 (Nov 2021–Jan 2022)	1	251	221	157	0	5	71.04
Round 11 (Feb–Apr 2022)	6	257	214	143	0	1	66.82
Total:	257	257	NA	1347	8	29	62.77

* Enrolment and data collection paused due to the COVID-19 pandemic.

**Table 2:** Characteristics of study sample at baseline.

	*n* (%)
Total	256 (100%)
Gender (*n* = 256)	
Cisgender man	145 (57%)
Cisgender woman	101 (39%)
Transgender, gender diverse, or two-spirit	10 (3.9%)
Race/Ethnicity (*n* = 250)	
Indigenous (Aboriginal, First Nations, Inuit, Metis)	99 (40%)
Asian (Indian, Pakistani, Chinese, Vietnamese, Japanese, Filipino)	11 (4.4%)
Black (African, Caribbean)	7 (2.8%)
White (European or European descent)	122 (49%)
Not captured by above categories	11 (4.4%)
Born outside of Canada (*n*-255)	23 (9.0%)
Age (*n* = 256)	
Less than 45 years old	80 (31%)
45–60 years old	156 (61%)
60+ years old	20 (7.8%)
Relationship status (*n* = 255)	
Single/Dating	177 (69%)
Partnered/Married/Common law	63 (25%)
Separated/Divorced/Widowed	15 (5.9%)
Housing situation (*n* = 256)	
Homeless past 30 days	27 (11%)
Stably housed	229 (89%)
Educational attainment (*n* = 256)	
Less than high school	132 (52%)
High school or more	124 (48%)

**Table 3:** Lifetime and current substance use and health, *n* (%) or Median (IQR) (*n* = 256).

	Lifetime	Current ^a^
Substance use ^b^	254 (99%)	218 (86%)
Binge use	195 (78%)	72 (32%)
Daily opioid use ^c^	251 (98%)	69 (27%)
Daily stimulant use ^d^	245 (96%)	51 (20%)
Accidental overdose	140 (56%)	29 (12%)
Enrolled in substance use disorder treatment ^e^	--	127 (50%)
Satisfaction with health, range 0–10 (higher = higher satisfaction)	--	7.00 (6.00, 9.00)
WHO Disability Assessment Schedule, range: 0–48 (higher = greater functional impairment)	--	14 (6, 22)
Modified Colorado Symptom Index score, range: 0–50 (higher = higher symptom frequency)	--	12 (5, 19)

^a^ Refers to past 3 months for all substance use and treatment outcomes; past day for self-rated health; and, past 30 days for WHO-DAS, and Modified Colorado Symptom Index. ^b^ Includes heroin, crack cocaine, cocaine powder, amphetamine, speedballs (down unspecified and cocaine), goofballs (down unspecified and amphetamine), Dilaudid, morphine, fentanyl, prescription opioids, prescription stimulants, sedatives, ecstasy, ketamine, GHB, hallucinogens. ^c^ Includes heroin, fentanyl, and down (unspecified). ^d^ Includes cocaine, crack cocaine, and crystal meth. ^e^ Lifetime enrolment in substance use disorder treatment was not solicited.

**Table 4:** Economic engagement in total sample and by age group, *n* (%) or median (IQR) (*n* = 256).

	Lifetime	Current ^a^
Average monthly income ($ CAD) ^d^	--	1955 (1521, 2489)
Attended school/training program ^b^	202 (79%)	50 (20%)
Used employment services	141 (55%)	80 (31%)
Received income assistance		
Employable/hardship income assistance	200 (78%)	27 (11%)
Disability assistance	216 (84%)	202 (83%)
Employment insurance	97 (38%)	2 (0.8%)
Old age security/Public pension	22 (8.6%)	20 (7.8%)
Income generation		
Informal/prohibited/illegal activities	245 (96%)	191 (75%)
Recycling (binning, buy/sell) ^c^	153 (60%)	80 (31%)
Squeegeeing	18 (7.0%)	0 (0%)
Panhandling	73 (29%)	14 (5.5%)
Theft, stealing (shoplifting, breaking into cars/houses)	120 (47%)	17 (6.6%)
Selling needles	18 (7.0%)	1 (0.4%)
Selling cigarettes/tobacco	81 (32%)	28 (11%)
Selling drugs/enforcing	150 (59%)	43 (17%)
Sex work	21 (8.2%)	2 (0.8%)
Other criminal(ized) activity	90 (35%)	24 (9.4%)
Stipend	192 (75%)	152 (59%)
Formal employment	246 (96%)	129 (50%)
Self-employed	136 (53%)	50 (20%)
Primary employment-income source		
Casual (on-call, day labour)	--	105 (42%)
Temporary/fixed term contract	--	22 (8.8%)
Self-employed	--	20 (8.0%)
Permanent part-time (<30 h/week)/varied hours	--	88 (35%)
Permanent full-time (30 h or more/week)	--	14 (5.6%)
Labour force participation		
Always had formal job or was looking for work	16 (6.2%)	--
Usually have a job or looking for one	82 (32%)	--
Vary between working/looking for work and not working/not looking	78 (30%)	--
Rarely working or looking for work	66 (26%)	--
Have never had or looked for formal job	14 (5.5%)	--

^a^ Refers to past 3 months. ^b^ Does not include primary/secondary education, Current = currently enrolled or planning to enroll in school/training program. ^c^ Recycling activities involve the salvaging of recyclable materials in exchange for payment through municipal recycling programs. ^d^ Equivalized to 2022 value.

## Data Availability

Study data and the data dictionary will not be publicly available because of the sensitivity of distributing data gathered from disadvantaged participants engaged in criminalized behaviour. The sharing of data will be considered by application to the study principal investigator and corresponding author at bccsu-lr@bccsu.ubc.ca on a case-by-case basis. In cases where requests to access study data are approved, the data dictionary and deidentified participant data, including only variables relevant to the application, will be provided. Access criteria for the data include release only to researchers with investigator support after approval of a proposal with a signed data access agreement and upon proof of completion of the requisite data protection protocols.

## Appendix A

**Table A1:** Table of measures.

		BL	FUP	EXIT
**Demographics & Background**				
A1	Age	x		
A1	Ethnicity	x		
A2	Gender identity	x		
A3	Relationship status	x	x	x
A4	Immigration (place of birth)	x		
A6	Re-location	x	x	x
A6	Neighbourhood	x	x	x
A7	Co-habitation	x	x	x
A9	Housing type and stability	x	x	x
**Community Connectedness**				
B1-2	Community Involvement Measure	x	x	x
B3	Social Inclusion Scale	x	x	x
B4	Neighbourhood Cohesion	x	x	x
**Economic Participation & Employment**				
C2	Employment Precarity	x	x	x
C11	Unemployment/non-participation	x		
C4	Government assistance	x	x	x
C1	Income Generation (across continuum)	x	x	x
C3	Employment—formal & informal	x	x	x
C12	Disruptive Events	x	x	x
C9	Monthly Total Income, Allocation of monies	x	x	x
**Work Intentions and Barriers**				
D(1-6)	Perceptions of work: motivation to work, benefits of work (manifest and latent benefits)	x	x	x
D7	Barriers to work	x	x	x
D8	Perceived Employability Scale	x	x	x
D9	Latent functions of employment (time structure, financial strain,	x	x	x
D10	Resilience	x	x	x
**Material Security**				
E1	Material Security	x	x	x
E2	Food Security Scale	x	x	x
**Education and Training**				
F1-3	Education & Training (partial, completed, current, planned, referrals	x	x	x
F4	Training rigor	x	x	x
G1	Employment service engagement (accessed, referrals, programs)	x	x	x
G2	Barriers to accessing employment service	x	x	x
**Substance Use and drug-related harm**				
H1	Substance past 12 months	x		
H2	Substance past 3 months: drug, route, frequency, dose, street value	x	x	x
H4	Drug expenditure	x	x	x
H5	Reduced risk Substance Use Practices	x	x	x
H6	Riskier Substance Use Practices	x	x	x
H8	Reasons for Substance Use	x	x	x
H9	Substance use related to work	x	x	x
J1-5	Binge drug use (length, frequency, harms)	x	x	x
K1-7	Overdose (number, substance, route, help)	x	x	x
**Debt**				
L1	Debt (who, reasons)	x	x	x
L2-7	Drug debt and repercussions	x	x	x
**Health measures**				
M1	Violence exposure (Childhood, adulthood, current)	x	x	x
M3	Violence perpetration	x	x	x
N1	Satisfaction scale/quality of life	x	x	x
N2	Mental health	x	x	x
N3	Physical Health	x	x	x
N4	Current Health State	x	x	x
**Service Utilization and Service access barriers**				
O1-2	Health Care (Hospital Admissions, emerg dept, drug use Tx EMS & paramedics, use and barriers)	x	x	x
O3-4	Social Services (use and barriers)	x	x	x
O5	Criminal Justice System (police interactions, circumstances)	x	x	x
**COVID-19**				
CV1-2	Awareness and Testing	x		
CV12-14	Social Distancing and precautions	x	x	x
CV15-16	Housing precautions	x	x	x
C17	Community connectedness	x	x	x
C18-21	Education and training (enroll, barriers)	x	x	x
CV23-33	Income generation safety and changes	x	x	x
CV34-36	Social Assistance (changes, adequacy, barriers, clawbacks)	x	x	x
CV37-40	Substance use changes	x	x	x
CV41-42	Drug debt changes	x	x	x
CV43-44	Violence (social distancing, self-isolation, police presence)	x	x	x
CV46	Social services utilization barriers	x	x	x
CV47	Health services utilization barriers	x	x	x
CV48	Treatment services utilization barriers	x	x	x
CV49-54	Safe supply (use, adequacy, reasons barriers)	x	x	x

## Appendix B. Qualitative Enrolment and Follow-Up Schedule

**Table A2:** Quarterly and cumulative enrolment and follow-up for the qualitative sample from February 2021 to April 2022.

	Baseline Visits	Cumulative Enrolment	Eligible for Follow-up	Follow-up Visits	Withdrew	Deceased	Follow-Up Rate (%)
Round 07 (February–April 2021)	26	26	0	0	0	0	0
Round 08 (April–July 2021)	11	37	0	0	0	1	0
Round 09 (July–October 2021)	3	40	25	21	0	0	84
Round 10 (November 2021–January 2022)	1	41	15	8	0	0	53.33
Round 11 (February–April 2022)	0	41	31	26	0	1	83.87
Total	41	41	NA	55	0	2	73.73
